# Expression of long non-coding RNA H19 predicts distant metastasis in minimally invasive follicular thyroid carcinoma

**DOI:** 10.1080/21655979.2019.1658489

**Published:** 2019-09-06

**Authors:** Yan Dai, Yuanqing Miao, Qingli Zhu, Mei Gao, Fengyun Hao

**Affiliations:** a Department of Endocrinology, Linyi Central Hospital, Linyi, Shandong, China; b Department of Medical Network Information Center, the Affiliated Hospital of Qingdao University, Qingdao, Shandong, China; c Department of Thyroid surgery, The Affiliated Hospital of Qingdao University, Qingdao, Shandong, China; d Department of Pathology, The Affiliated Hospital of Qingdao University, Qingdao, Shandong, China

**Keywords:** Follicular thyroid carcinoma, metastasis, distant metastasis, prognosis, long non-coding RNA H19

## Abstract

Downregulation of lncRNA H19 (H19) expression is associated with an unfavorable prognosis in some cancers. However, little was known as to whether there was an association between H19 and minimally invasive follicular thyroid carcinoma (MI-FTC). In our study, we used quantitative real-time polymerase chain reaction (qRT-PCR) to determine H19 expression in 186 patients with MI-FTC who underwent initial surgery. Of the 186 patients with MI-FTC, 21 patients show distant metastasis （M+）at the initial operation established the diagnosis of MI-FTC. Of the 165 patients who did not show distant metastasis at diagnosis during the follow-up period (≥10 years), 28 patients undergone M+ and 137 patients has no distant metastasis（M-）after the initial operation. Low H19 expression was associated with large tumor size, vascular invasion, and distant metastasis. Univariate analysis showed that gender (male), age (45 years or older), primary tumor size (4 cm or more), vascular invasion and H19 level (<1.12) were significant prognostic factors related to postoperative distant metastases. Multivariate analysis showed that age, primary tumor size (4 cm or more) and vascular invasion was a significant prognostic factor for survival. Patients with low H19 expression showed a poorer outcome in MI-FTC patients. Receiver-operating characteristic (ROC) curve analysis demonstrated H19 could distinguish M+ from M- patientswith a value of area under the curve (AUC). Our findings suggest that H19 is a potential prognostic factor for evaluating prognosis and the metastatic potential of MI-FTC at an initial operation stage.

## Graphical Abstract

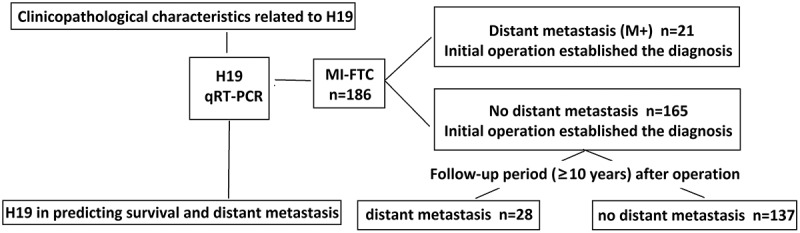

## Introduction

Follicular thyroid cancers (FTC) comprise between 10–15% of all differentiated thyroid cancers. According to the World Health Organization (WHO) classification of thyroid tumors, FTC is defined by the presence of capsular and/or vascular invasion and by the absence of nuclear features typical of papillary thyroid carcinoma (PTC) [1]. FTC is more likely to metastasize to distant organs rather than to regional lymph nodes because of its tendency to invade blood vessels thus resulting in hematogenous dissemination [2]. Like PTC, FTC retains many characteristics of thyroid follicular cells, including the expression of thyroid-specific proteins such as TSH receptor, thyroid peroxidase (TPO), and thyroglobulin (Tg) that serve as targets for thyroid autoimmunity. Despite the theoretically specific definition of FTC, diagnostic controversy and interobserver variability make the diagnosis of FTC difficult. FTC is further divided into minimally invasive FTC (MI-FTC), which only has limited capsular and/or vascular invasion and widely invasive FTC (WI-FTC), which exhibit widespread infiltration of thyroid tissue and/or vascular invasion [2]. Several authors usually reported a benign clinical course of MI-FTC owing to the low risk of tumor recurrence and distant dissemination [3,4]. Conversely, other authors described that also MI-FTCs can give rise to distant metastases [5]. Both types, like most well-differentiated thyroid cancers, are traditionally treated the same: a completion thyroidectomy usually followed by radioiodine ablation. MI-FTC shows good long-term outcomes. However, in some cases, MI-FTC metastasizes to the lung and bone, exhibiting a poor prognosis (i.e., metastatic MI-FTC, MI+). Nonetheless, distinguishing between metastatic and non-metastatic MI-FTCs (MI-) is currently difficult by any pathological modalities. When distant metastasis is recognized during the follow-up period, additional therapies, such as completion total thyroidectomy and radioiodine ablation therapy, are needed [6]. Thus, identification of prognostic biomarkers for predicting groups at high risk for metastasis among patients diagnosed with MI-FTC after the initial operation should be important in the postoperative follow-up of MI-FTC.

Studies have suggested that age, sex, tumor size, and/or vascular invasion are clinicopathological risk factors for distant metastasis of MI-FTC. Currently accepted the histological classification of FTC is inadequate and fails to accurately predict patients with distant metastatic disease and a more aggressive clinical course [7]. Thus, to date, risk factors for predicting aggressive tumor behavior and distant metastasis remain unclear and there has been a growing interest to discover risk factors of distant metastasis through molecular biological research. The long noncoding RNA H19 (H19), which is 2.3 kb in length and located in chromosome 11, is a nonprotein-coding imprinted and maternally expressed lncRNA [8]. Aberrant expression of H19 has been associated with tumor progression [9,10]. We have recently found that expression of H19 was lower in the papillary thyroid carcinoma (PTC) tissues, and low expression of H19 was associated with extrathyroid extension, pathological lateral node metastasis, histological aggressive type and poorer disease-free survival (supplementary file).

In this study, we aimed to identify H19 in predicting prognosis of patients with MI-FTC in samples of MI-FTC obtained at the initial operation using a quantitative PCR-based array. Furthermore, we assessed the potential use of H19 as novel biomarkers for the distant metastasis and patient prognosis.

## Materials and methods

### FTC specimens

One hundred and eighty-six patients with MI-FTC who underwent surgery between 2001 and 2008. The 186 cases met the following criteria: (i) histopathological evaluation of the primary surgical specimens as MI-FTC was done according to the criteria of World Health Organization, (ii) patients had undergone neck ultrasonography routinely for ≥10 years after surgery. Patients with atypical follicular adenomas, WI-FTC, follicular variant of PTCs, or poorly differentiated (insular) tumors were excluded. The type of surgery was registered as total thyroidectomy or lobectomy, where supplementary lobectomy within a month after the original operation was classified as a total thyroidectomy. The medical records of these patients were retrospectively reviewed, and their clinicopathological characteristics and outcome data were recorded. Written informed consent was not obtained from participants for their clinical records and tissues to be used in this study. The present study was conducted in accordance with the ethical standards of the Helsinki Declaration in 1975, after approval of the Institutional Review Board of the affiliated hospital of Qingdao University(2014-ES-028). We categorized 186 patients with MI-FTC into two groups: the distant metastases (M+) (n = 49) and the non-metastatic group M−(n = 137). In the M (+) group, distant metastasis was diagnosed at the time of the initial surgery (n = 21) and after the initial operation established the diagnosis of MI-FTC during the follow-up period (n = 28). In the M (−) group, no distant metastasis was recognized postoperatively for ≥10 years. Although patients in both groups were clinicopathologically diagnosed with MI-FTC at the time of the initial operation, neither routine pathological examination nor clinical data could distinguish between the M (+) and M (−) groups. Clinical characteristics of the patients are presented in Table 1.

**Table 1.** Summary of clinicopathological features of the MI-FTC patients in this study.

Group		M+(n = 49)	M- (n = 137)	p-Value
**Sex (Femal/Male)**		26/23	121/16	<0.05
**Age (years)**		58.4 ± 14.6	40.6 ± 13.8	<0.05
**Tumor size(mm)**		47.7 ± 11.5	30.8 ± 9.6	<0.05
**Operation method**				0.0000
Right or left thyroid lobectomy		8	72	
Total thyroidectomy		41	65	
**Invasion**				
Capsular invasion		18/31	106/31	NS
Vascular invasion		34/15	63/74	<0.05
**Distant metastasis before surgery (n = 21)**				
Location	Lung	15		
	Bone	6		
	Others	3		
**Distant metastasis after surgery (n = 28)**				
**Location**	Lung		21	
	Bone		7	
	Others		3	
Period (month)			4.1 ± 1.7	
**Nodal metastases**				NS
Yes		9	16	
No		40	121	
**Administration of I**^131^				<0.05
Yes		43	98	
No		6	39	
**External beam irradiation**				<0.05
Yes		15	16	
No		34	121	

### miRNA extraction and RT-qPCR analysis

Total miRNA was isolated from frozen tissues using a mirVana miRNA isolation kit (Ambion). Expression of H19 was validated using TaqMan miRNA assays (Applied Biosystems). Briefly, 10 ng total RNA was reverse-transcribed using a reverse transcription (RT) primer specific for H19 with MultiScribe Reverse Transcriptase (Applied Biosystems). The primers used for H19 was 3ʹ-TACAACCACTGCACTACCTG-5ʹ, 5ʹ-3ʹTGGCCATGAAGATGGAGTCG. The RT products were subsequently subjected to a PCR reaction with primer sets specific for H19. Amplification of miRNA-derived PCR products was monitored on an ABI 7300 Real-Time PCR system (Applied Biosystems). All reactions were performed in triplicate and RNU6B was used as a reference for data normalization. The relative expression of H19 was calculated as 2^−(ΔCt-ΔCNAT)^.

### Statistical analysis

Data were presented as mean ± standard deviation (SD) from at least three separate experiments. Mann–Whitney U and Kruskal–Wallis analyzes of variance were used to evaluate statistical differences in tissue H19 expression between unpaired groups and multiple comparison groups, respectively. The t-test, chi-squared and ANOVA were used to measure differences in H19 expression levels in different subgroups based on clinical/pathological variables. A comparison of the diagnostic abilities for H19 was performed using the area under the curves (AUC). Survival was evaluated at the end of follow up using the Kaplan-Meier analysis. To assess the prognostic value of H19 in the prediction of metastasis after the initial MI-FTC operation, odds ratios (ORs) with 95% confidence intervals (CIs) were calculated. Either the χ2 test or the Mann–Whitney U test was used to examine a possible association between metastatic status and clinicopathological parameters including H19. Only variables that were significant in univariate analyses were used in a multivariate model. The reported results included HR and 95% confidence intervals (CI). All statistical analyses were performed with SPSS 13.0 software. p < 0.05 was considered significant.

## Results

### Baseline characteristics of study subjects

Baseline characteristics of the 186 FTC patients in our study cohort are listed in Table 1. The mean age of patients in the M+ group (n = 49) was (58.4 ± 14.6) years [66 years (range; 45–79)], and the female was 39. 41 M+ patients received a total thyroidectomy and 8 patients received right or left thyroid lobectomy. The mean tumor size was (47.7 ± 11.5) cm [52 mm (range, 25–150 mm). 41 and 34 patients of M+ had capsular or vascular invasion, respectively. Twenty-four patients have distant metastasis before surgery (lung 15, bone 6, others 3); 9 of 49 M+ patients had cervical lymph nodes metastasis. Forty-three patients received the administration of I^131^ and 19 patients received external beam irradiation.

In the M- groups(n = 137), the median time between initial surgery and detection of metastasis was (4.1 ± 1.7) [3.2(1.8–8.3)] years in these patients. The mean age of M- was (40.6 ± 13.8) years [45.6 years (range; 29–77)], and the female was 109 and 65 patients received a total thyroidectomy and 72 patients received right or left thyroid lobectomy. The mean tumor size was (30.8 ± 9.6) mm [32 mm (range, 18–84 cm). 98, 39 patients had capsular or vascular invasion, respectively. Thirty-one patients have distant metastasis after surgery (lung 21, bone 7, others 3); 16 of 137 patients had cervical lymph nodes metastasis. Ninety-eight patients received the administration of I^131^ and 16 patients received external beam irradiation.

The patients' age and tumor size in M+ group was higher and larger compared with the M- group (P = 0.024, P = 0.043). Most of the patients in M+ group has a vascular invasion, and receive total thyroidectomy, administration of I^131^ and external beam irradiation, (P = 0.033, P = 0.000024, P = 0.037 and P = 0.0047, respectively). No significant relation was found between patient’s capsular invasion (P = 0.137) and nodal metastases (P = 0.582), respectively.

### Clinicopathological characteristics related to H19 in MI-FTC

qRT-PCR was conducted to determine the expression levels of H19 **in** patients with MI-FTC. The 186 patients were separated into H19 high risk (higher levels) and low risk (lower levels) groups using Cutoff Finder software (http://molpath.charite.de/cutoff), to generate the optimum cutoff score for the normalized serum expression (-Δ^Cq^). The clinicopathological features of the MI-FTC included in our study are summarized in Table 2. We found that low H19 expression was significantly related with tumor size (p = 0.018), vascular invasion (p = 0.004) and distant metastasis (p = 0.035 and p = 0.012). No significant relation was found between gender (p = 0.476), age (p = 0.073), lymph node metastasis (p = 0.246), administration of I^131^ (p = 0.436) and external beam irradiation (p = 0.184).

**Table 2.** Analysis of clinical characteristics and H19 expression related to patients with MI-FTC.

Group	Total number	H19 levels	*p*-value
**Gender**			0.476
Femal	152	1.22(0.76–2.34)	
Male	34	1.25(0.68–2.28)	
**Age (year)**			0.073
≥45	107	1.08(0.68–2.14)	
<45	79	1.21(0.76–2.41)	
**Tumor size(cm)**			0.018
≥4	82	0.96(0.73–2.06)	
<4	104	1.21(0.82–2.43)	
**Invasion**			0.004
Capsular invasion	98	1.23(0.68–2.38)	
Vascular invasion	103	0.97(0.76–2.19)	
**Lymph node metastasis**			0.246
Yes	25	1.09(0.69–2.37)	
No	161	1.15(0.82–2.32)	
**Distant metastasis before diagnosis**			0.035
M+	24	1.03(0.68–2.31)	
M-	162	1.23(0.84–2.24)	
**Distant metastasis after diagnosis**			0.012
M+	31	0.93(0.71–2.07)	
M-	131	1.21(0.76–2.39)	
**Administration of I**^131^			0.436
Yes	141	1.13(0.78–2.37)	
No	45	1.21(0.63–2.24)	
**External beam irradiation**			0.184
Yes	30	1.09(0.68–2.28)	
No	156	1.20(0.79–2.39)	

### Clinicopathological characteristics related to distant metastasis in patients after operation

Univariate analysis showed that age [(>45 years; OR (95% CI): 4.83 (1.03–20.5),p = 0.026], male sex [OR (95% CI):3.24 (0.96–14.7), p = 0.048), tumor size >4 cm [OR (95% CI):5.17 (1.13–28.4),p = 0.012], Vascular invasion[OR (95% CI): 7.14 (1.42–25.6),p = 0.002] and low H19 level [OR (95% CI): 6.33 (1.12–21.5),p = 0.018]were significant factors related to distant metastases (Table 3). Multivariate analysis showed that age [(>45 years; OR (95% CI): 4.73 (0.98–18.3), p = 0.036], tumor size >4 cm [OR (95% CI): 4.74 (1.04–21.7), p = 0.04] and vascular invasion [OR (95% CI): 6.32 (1.24–23.3), p = 0.014] was the independent risk factor related to distant metastasis after operation (Table 3).

**Table 3.** Univariate and multivariate analysis of risk factors for distant metastasis in patients after operation.

Groups	Without metastasis	Metastasis		Univariate		Multivariate	
Gender	M-(n = 137)		M+(n = 28)	OR (95% CI)	p	OR (95% CI)	p
Femal vs Male	121/16		15/13	3.24 (0.96–14.7)	0.048	2.76 (0.82–10.4)	0.143
**Age (year)**							
<45 vs ≥45	60/77		8/20	4.83 (1.03–20.5)	0.026	4.73 (0.98–18.3)	0.036
Tumor size(cm)							
<4 cm vs ≥4 cm	84/53		10/18	5.17 (1.13–28.4)	0.012	4.74 (1.04–21.7)	0.04
**Operation method**							
Hemi. vs Total	57/80		12/16	2.84 (0.72–17.34)	0.19		
**Capsular Invasion**							
Yes vs No	86/51		15/13	3.05 (0.78–18.67)	0.095		
**Vascular Invasion**							
Yes vs No	43/94		16/12	7.14 (1.42–25.6)	0.002	6.32 (1.24–23.3)	0.014
**Lymph node metastasis**							
Yes vs No	78/59		17/11	3.26 (0.68–16.53)	0.196		
**Administration of I**^131^							
Yes vs No	87/50		16/12	2.87 (0.56–10.62)	0.431		
**External beam irradiation**							
Yes vs No	76/61		14/14	2.35 (0.52–9.65)	0.524		
**H19 level**							
<1.12 vs ≥1.12	44/93		15/13	6.33 (1.12–21.5)	0.018	3.53 (0.72–18.53)	0.103

### Significance of H19 in predicting survival and distant metastasis during the ≥10-year follow-up

**Figure 1.**
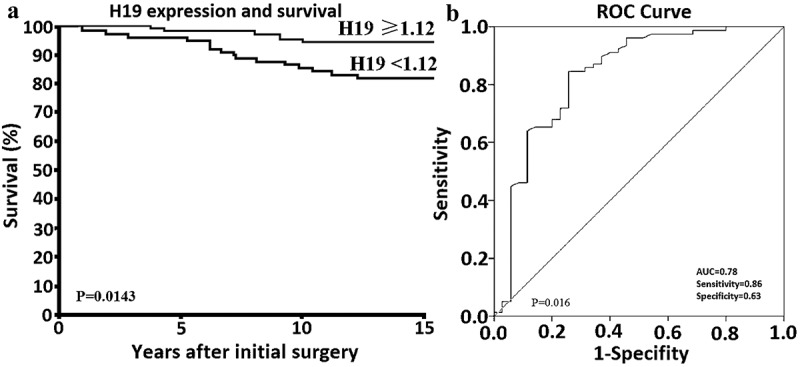
Expression of H19 in MI-FTC and its diagnostic and prognostic values. (a) Receiver operating characteristic (ROC) curves for H19 levels for reflecting distant metastasis and non-distant metastasis in 165 patients after the initial operation established the diagnosis of MI-FTC during the follow-up period. (b) Kaplan–Meier plot illustrating overall survival related to H19 expression among the 186 MI-FTC patients.

Based on the ROC curves constructed, H19 at the cutoff value of 1.12 had good performance for differentially predicting M+ versus M- with an area under (AUC of 0.78; sensitivity 0.86; specificity 0.63) (P = 0.016) (Figure 1). The Kaplan-Meier analysis and the log-rank test were used to calculate the effect of H19 on patient survival (Figure 1). The median follow-up period was 108.6 months (range, 15–208) and the median time to distant metastasis was 38.4 months (range, 14–128 months). Specifically, the mean overall survival (OS) time of patients who has low levels of H19 (<1.12 relative expression value) was 89 months, whereas the mean OS time of those with high levels of H19 was 166 months. A significant association between a low H19 level and poor OS (HR 2.68; 95% CI: 1.03 to 7.8); P = 0.0143) was found.

## Discussion

MIFTC is confirmed on pathological examination only after diagnostic hemithyroidectomy and it is difficult to preoperatively determine whether patients require complete thyroidectomy. Because of its excellent prognosis, it is generally known that MIFTCs do not need completion thyroidectomy [11]. Some patients at risk of developing distant metastasis, however, require complete thyroidectomy and radioactive iodine (RAI) ablation [12]. This strategy allows detection of otherwise invisible metastases by whole-body scintigraphy and thyroglobulin measurements, as well as early detection of recurrent disease. This strategy allows detection of otherwise invisible metastases by whole-body scintigraphy and thyroglobulin measurements, as well as early detection of recurrent disease. Although novel data may aid in the preoperative decision-making, we today have no sufficient tools to discriminate those patients that would benefit from more extensive treatment, although new molecular panels for fine needle aspirates have been proposed [13]. Still, several clinical features should be taken into account for the individual patient [14].

Of the 186 MI-FTC patients in our study cohort, 49 patients undergone distant metastasis and 137 patients did not undergo distant metastasis. Among these patients, the male, those aged ≥ 45 years, vascular invasion and tumor size >4 cm tended to be more easily distant metastasis. No significant difference was found between nodal metastases in distant metastasis group and non-distant metastasis group.

Recently, some studies have reported that H19 is a tumor suppressor in PTC cells [15,16], but other study has reported that H19 is a tumor promotor in PTC cells [17]. Therefore, its expression and role in patients with thyroid cancer was still unclear. In the present study, assessment of the correlation between expression of H19 and clinicopathological characteristics showed that H19 was less expressed in patients whose primary tumor size was 4 cm or more, vascular invasion, distant metastasis before or after diagnosis. No significant relation was found between gender, age, lymph node metastasis, administration of I131 and external beam irradiation and H19 expression. Therefore, the low H19 expression was significantly associated with the distant metastasis.

FTC frequently metastasizes to distant organs. Some FTCs initially present with distant metastasis, and the percentage of FTC patients with distant metastasis at initial diagnosis varies between 3.1% and 21% in the literature [18–20]. Distant metastasis is the most important prognostic factor in FTC. In the present study, 21 patients were with distant metastasis at initial diagnosis. Of the 165 patients without distant metastasis at initial diagnosis, 28 patients undergone distant metastasis, and 137 patients did not undergo distant metastasis during over 10 years follow-up observation. Our results were similar to the Sugino K’ et al. [21].

Several other prognostic indices have been proposed for MIFTC, such as male sex, older age, and/or large tumor size, which were associated with poor prognosis. In our study, univariate analysis showed that sex (male), age (45 years or older), primary tumor size (4 cm or more), vascular invasion and low H19 levels were significant prognostic factors related to postoperative distant metastases in the group of 165 patients without distant metastases at time of the initial surgery. Multivariate analysis showed that age, tumor size and vascular invasion was an independent risk factor for distant metastasis in patients with MI-FTC. Therefore, the prognoses of patients older than 45 years old, primary tumor size (>4 cm), vascular invasion and low H19 levels were poor and distant metastases often occurred. Routine completion total thyroidectomy and radioiodine ablation is thought necessary for these patients.

## Conclusions

In summary, low H19 levels were significantly associated with distant metastases and poor prognosis in MI-FTC patients. H19 is a potential prognostic factor for evaluating prognosis and the metastatic potential of MI-FTC at an initial operation stage.

## Article highlights

1. H19 is low expressed in follicular thyroid cancer(FTC) tissues
2. Low H19 expression was associated with vascular invasion and distant metastasis.
3. Patients with low H19 expression showed a poorer outcome.
