# Supplementary figures and images for: Expression of long non-coding RNA H19 predicts distant metastasis in minimally invasive follicular thyroid carcinoma
Source: Bioengineered. 2019 Sep 6;10(1):383–9. doi: 10.1080/21655979.2019.1658489 (PMC6738450; doi:10.1080/21655979.2019.1658489)

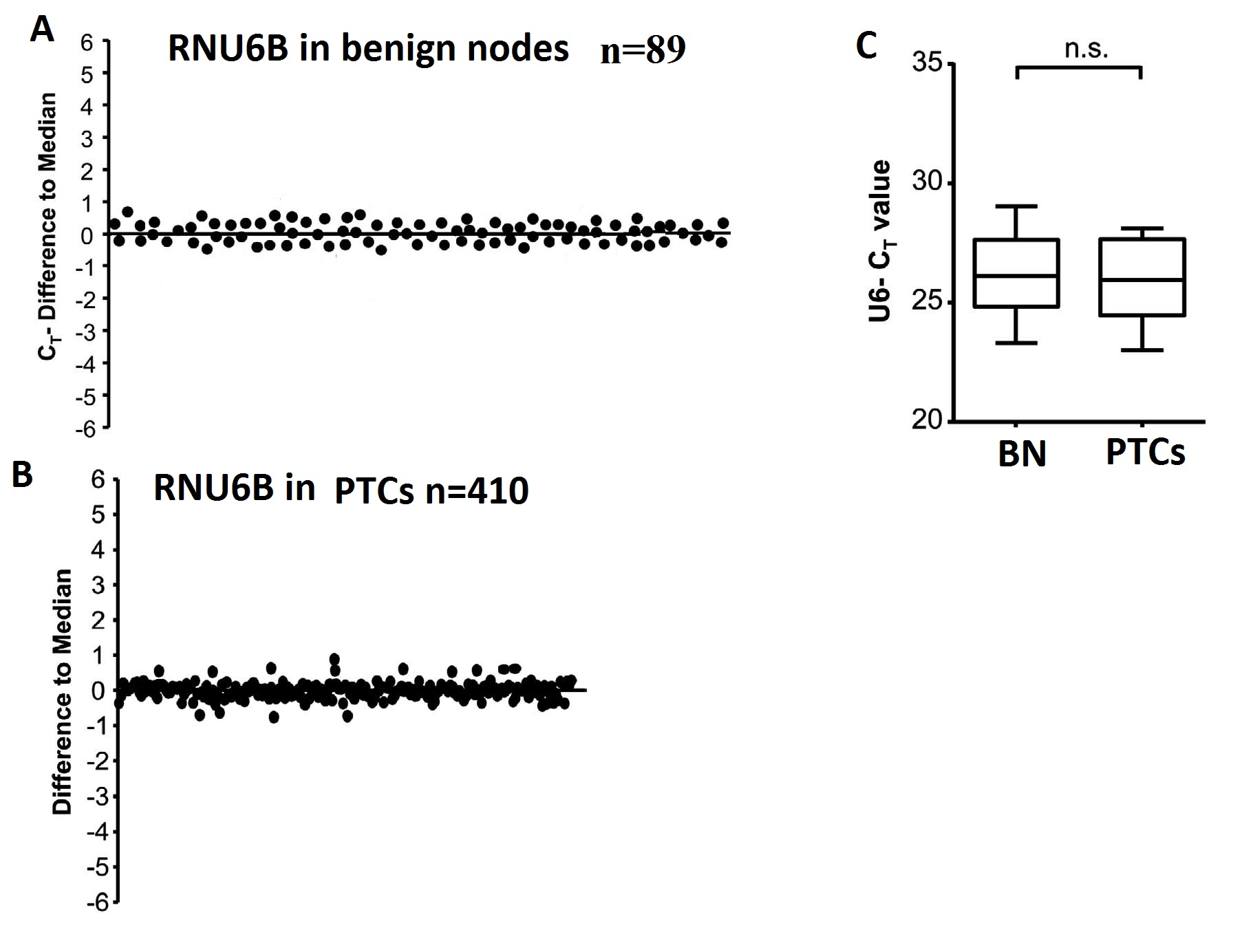

Supplement: Supplemental Material [file kbie-10-01-1658489-s001.jpg]
