# Supplementary material for: Expression of long non-coding RNA H19 predicts distant metastasis in minimally invasive follicular thyroid carcinoma
Source: Bioengineered. 2019 Sep 6;10(1):383–9. doi: 10.1080/21655979.2019.1658489 (PMC6738450; doi:10.1080/21655979.2019.1658489)
Supplement: Supplemental Material [file kbie-10-01-1658489-s002.pdf]

Cancer Biomarkers -1 (2019) 1–5 1
DOI 10.3233/CBM-190273
IOS Press

Long non-coding RNA H19 may be a marker for prediction of prognosis in the follow-up of patients with papillary thyroid cancer

Xuelong Jiao[a], Jie Lu[b], Yichuan Huang[c], Jinna Zhang[b], Hongmei Zhang[b] and Kejun Zhang[b,*]
[a]*Department of General Surgery, The Affiliated Hospital of Qingdao University, Qingdao, Shandong, China*
[b]*Department of Thyroid Surgery, The Affiliated Hospital of Qingdao University, Qingdao, Shandong, China*
[c]*Department of Otolaryngology, The Affiliated Hospital of Qingdao University, Qingdao, Shandong, China*

Abstract.
OBJECTIVE: To investigate the diagnostic and prognostic values of long non-coding RNA H19 (H19) in patients with papillary thyroid carcinoma (PTC).
METHODS: This retrospective, nonrandomised study included 410 patients with PTC and 89 patients with benign thyroid nodes (BTN)who underwent standard total thyroidectomy. Real-time quantitative polymerase chain reaction (RT-qPCR) was used to detect H19 expression in these tissues. The relationship between H19 expression and the patients' clinicopathological factors, including histopathological characteristics of the tumour, diagnosis and prognosis was explored.
RESULTS: Expression of H19 was lower in the PTC tissues (1.259 ± 1.15) compared to the BNT tissues (2.8347 ± 2.176) ($p = 0.001$). Low expression of H19 was associated with patient's age, tumor size, extrathyroid extension, pathological lateral node metastasis (pN1b), histological aggressive type and poorer disease-free survival ($p < 0.0001$). The sensitivity for distinguishing PTC from benign was 81.3%. H19 was found to be an independent risk factor for extrathyroidal extension, lymph node metastasis.
CONCLUSIONS: H19 may serve as a potential predictor of poor prognoses in patients with PTC.

Keywords: Papillary thyroid carcinoma, diagnosis, prognosis, long non-coding RNA H19

1. Introduction

Thyroid cancer is a rare malignancy and accounts for less than 1% of malignant neoplasms in humans; however, it is the most common cancer of the endocrine system and responsible for most deaths from endocrine cancer. Thyroid cancer is histologically classified into differentiated thyroid cancer (DTC) (follicular thyroid cell-derived papillary thyroid cancer (PTC) and follicular thyroid cancer (FTC) poorly differentiated thyroid cancer (PDTC) and anaplastic thyroid cancer (ATC), and para-follicular C cell-derived medullary thyroid cancer (MTC), which classically account for approximately 80, 10, 5, 2 and 3% of all thyroid malignancies [1].

Although PTC is highly curable in general, approximately 10% of patients are destined for a progressive disease course with aggressive tumor behaviors and high disease recurrence and mortality rates [2–5]. This wide spectrum of disease behaviors often creates dilemmas in clinical risk stratification and decision making for the management of PTC. The aggressive group of PTCs poses a particularly difficult prognostic and therapeutic challenge. It has been suggested that novel molecular-based management would help tackle this challenge [6]. But the molecular mechanisms, particularly the genetic backgrounds, for the aggressiveness of this special group of PTCs remain to be better defined.

Long non-coding (Lnc) RNAs are defined as noncoding transcripts that are more than 200 nucleotides

*Corresponding author: Kejun Zhang, Department of Thyroid Surgery, The Affiliated Hospital of Qingdao University, Qingdao, Shandong, China. E-mail: thyroid_molecule@126.com.

ISSN 1574-0153/19/$35.00 © 2019 – IOS Press and the authors. All rights reserved

in length. Their expression deregulation plays an important role in the progress of cancer. The long non-coding RNA (lncRNA) H19 can function either as a tumor promoter or a tumor suppressor, depending on the type of cancer, development stage, or molecular background [7,8]. It is expressed in the embryo, down-regulated at birth and then reappears in tumors. Its role in tumor initiation and progression has long been a subject of controversy, although accumulating data suggest that H19 is one of the major genes in cancer [9]. It is actively involved in all stages of tumorigenesis and is expressed in almost every human cancer. Although H19 is a tumor suppressor in PTC cells [10,11], its expression and role in patients with thyroid cancer was still unclear.

In the present study, we detected the expression of H19 by qRT-PCR in tissues of PTC. We evaluated the prognosis of H19 expression in patients with PTC. This study reveals the differentially expressed H19 in human thyroid cancer, which may provide useful candidates for thyroid cancer in prediction of prognosis.

2. Materials and methods

2.1. PTC specimens

Specimens were collected from our frozen tumor tissue bank including 410 frozen PTC tissue and 89 patients with benign thyroid nodes who underwent thyroidectomy at the affiliated hospital of Qingdao University between January 2006 and December 2008. All of them had PTC by WHO criteria, while 57 of them showed aggressive features including tall cell variant, columnar cell variant, diffuse sclerosing variant, and presence of focal poorly differentiated features. There were 357 females and 53 males from 14 to 81 years of age (mean 46.4, median 48.6). Distant metastases were detected in 13 patients at the time of surgery who were classified as M1 in the UICC TNM classification system. Radioiodine had been administered to 128 patients, including patients with distant metastases as a treatment or to ablate possible thyroid remnants. Metastatic foci were detected with radioiodine scintigraphy in 14 patients, and radioiodine treatments were repeated in these patients. The patients were followed for a median of 86 months. In this series, when carcinoma recurrence was detected on imaging studies other than radioiodine scintigraphy, the patient was regarded as having a recurrence.

Table 1
Relationship between the H19 and clinicopathological features in 410 PTC patients

	Total number	H19 levels median (range)	p-value
Gender			0.189
Femal	357	0.98 (0.83–1.72)	
Male	53	0.99 (0.87–1.93)	
Age (year)			0.042
$\geqslant 60$	128	0.94 (0.78–1.73)	
< 60	282	1.13 (0.92–1.96)	
Tumor size (cm)			0.012
$\geqslant 4$	82	0.93 (0.84–1.70)	
< 4	328	1.16 (0.94–1.98)	
Extrathyroid extension			0.036
No or minimal	283	1.08 (0.87–1.92)	
Significant	127	0.93 (0.82–1.84)	
cN status			0.085
cN0 or cN1a	316	0.97 (0.82–1.89)	
cN1b	94	0.98 (0.86–1.94)	
M status			0.183
M0	391	0.99 (0.78–1.85)	
M1	19	0.97 (0.73–1.82)	
pN status			0.026
pN0 or pN1a	216	1.13 (0.87–1.89)	
pN1b	194	0.92 (0.85–1.78)	
Histological type			0.002
Common type	353	1.19 (0.98–2.03)	
Aggressive type	57	0.87 (0.72–1.58)	
Radioiodine			0.145
Yes	128	0.97 (0.74–1.82)	
No	282	0.98 (0.87–1.84)	
Recurrences			0.105
Yes	69	0.96 (0.76–1.88)	
No	341	0.98 (0.84–1.92)	

2.2. miRNA extraction and RT-qPCR analysis

Total miRNA was isolated from frozen tissues using a mirVana miRNA isolation kit (Ambion). Expression of H19 was validated using TaqMan miRNA assays (Applied Biosystems). Briefly, 10 ng total RNA was reverse-transcribed using a reverse transcription (RT) primer specific for H19 with MultiScribe Reverse Transcriptase (Applied Biosystems). The primers used for H19 was 3'-TACAACCACTGCACTACCTG-5', 5'-3'TGGCCATGAAGATGGAGTCG. The RT products were subsequently subjected to a PCR reaction with primer sets specific for H19. Amplification of miRNA-derived PCR products was monitored on an ABI 7300 Real-Time PCR system (Applied Biosystems). All reactions were performed in triplicate and U6 small nuclear 2 RNA (RNU6B) was used as a reference for data normalization. The relative expression of H19 was calculated as $2^{-(\Delta \mathrm{Ct}-\Delta \mathrm{CNAT})}$.

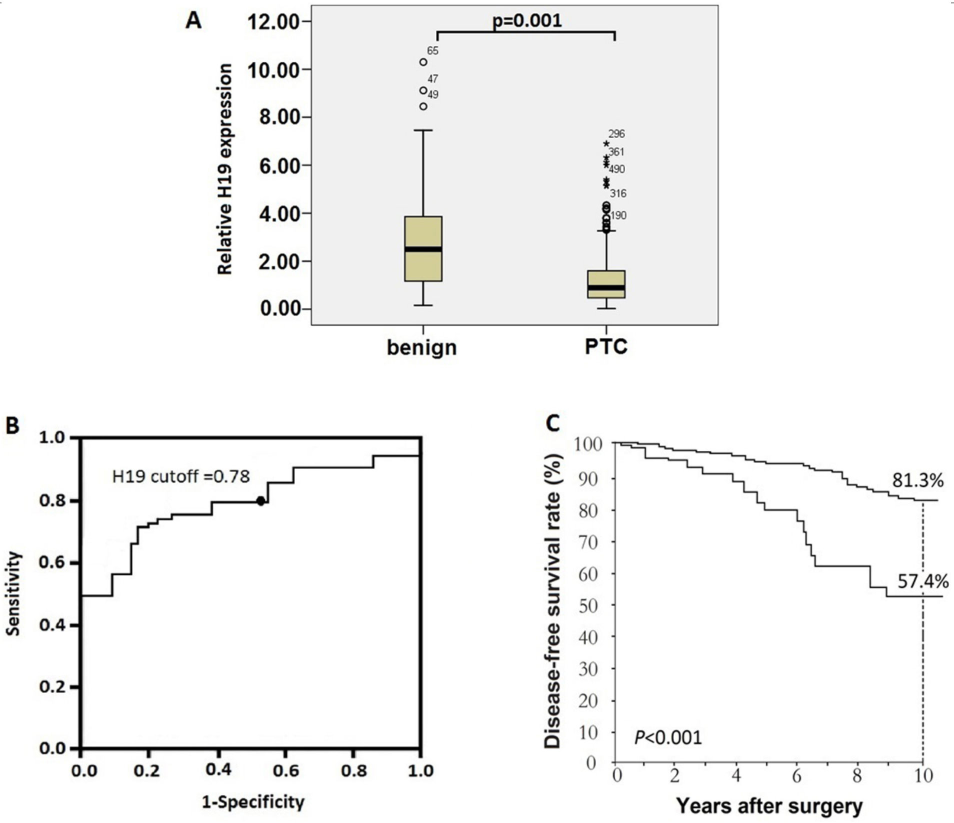

Fig. 1. Expression of H19 in PTC and its diagnostic and prognostic values. A, Expression of H19 in PTC and BTN tissues. $P = 0.001$, Mann-Whitney U assay. B, Receiver operating characteristic (ROC) curves for H19 levels for reflecting PTC and benign nodes. C, Kaplan-Meier curves of disease-free survival by H19 levels.

2.3. *Statistical analysis*

Data were presented as mean ± standard deviation (SD) from at least three separate experiments. Mann–Whitney U and Kruskal–Wallis analyses of variance were used to evaluate statistical differences in tissue H19 expression between unpaired groups and multiple comparison groups, respectively. The t-test, chi-squared and ANOVA were used to measure differences in H19 expression levels in different subgroups based on clinical/pathological variables. A comparison of the diagnostic abilities for H19 was performed using the area under the curves (AUC). Survival was evaluated at the end of follow up using the Kaplan-Meier analysis. Univariate and multivariate analyses were performed for prognostic factors of survival using the Cox regression model. Only variables that were significant in univariate analyses were used in a multivariate model. The reported results included HR and 95% confidence intervals (CI). All statistical analyses were performed with SPSS 13.0 software. $p < 0.05$ was considered significant.

3. Results

3.1. *H19 is downregulated in PTC tissues*

We had proven that RNU6B was stably expressed in PTCs and benign thyroid nodes (BTN) tissues (data not shown), so we used RNU6B as the reference gene. To investigate the expression level and significance of H19 expression in PTCs, we performed quantitative real-time PCR (qRT–PCR) to evaluate H19 expression in 410 PTC tissues and 89 benign thyroid nodes (BTN) tissues. The results showed that H19 expression was significantly downregulated in PTC tissues (1.259 ± 1.15) compared with BTN tissues (2.8347 ± 2.176) ($p = 0.001$) (Fig. 1A).

3.2. *H19 and its relationship with clinicopathological features in patients with PTC*

We compared the H19 expression and clinicopathological features of the 410 PTC patients (Table 1). We

Table 2
Univariate and multivariate Cox regression analysis of factors associated with prognosis of patients with PTCs

Variable ($n = 410$)		Univariate analysis Hazard ratio (95% CI)	p	Multivariate analysis Hazard ratio (95% CI)	p
Age (years)	> 60	2.4 (1.73–5.15)	0.006	3.84 (1.38–10.47)	0.019
Tumor size (cm)	> 4	2.3 (1.10–4.80)	0.026	2.18 (0.87–3.14)	0.086
Histological type	Aggressive type	3.18 (1.39–7.88)	0.007	2.75 (0.783–6.36)	0.127
cN status	cN1b	2.32 (0.96–4.17)	0.028	1.45 (0.83–3.50)	0.116
pN status	pN1b	1.84 (1.02–3.57)	0.102		
Extrathyroid extension	Significant	2.48 (1.29–4.93)	0.001	2.36 (1.16–4.07)	0.025
H19	< 0.78	1.89 (1.02- 3.54)	0.021	1.92 (1.16–4.18)	0.038

found that the H19 expression was associated with patient's age, tumor size, extrathyroid extension, pathological lateral node metastasis (pN1b) and histological aggressive type of PTC. But not with patients' gender, radioiodine, cN status, M status and recurrences.

3.3. *H19 expression and prognosis in PTC patients*

During the study period, of the 410 PTC patients, 69 patients developed locoregional recurrences, 19 patients developed distant metastases, 10 of these patients had both locoregional recurrence and distant metastases, and 10 patients died of the disease. The patients underwent PTC resection from 2006 to 2009 and their 10-year follow-up information was available for each patient. The optimal cutoff value of H19 producing maximum sensitivity plus specificity for reflecting the presence of PTC from the ROC analysis was 0.78. The ROC curve analysis using H19 levels yielded an AUC of 0.784 (95% CI: 0.65 to 0.90; $P < 0.001$) in discriminating PTC from benign controls as shown in Fig. 1A. As to the cutoff value, the number of PTC patients with H19 > 0.78 was 134, and 276 patients with H19 value $\leqslant 0.78$. Sensitivity and specificity were 81.3% and 52.8% (Fig. 1B).

Low H19 expression was significantly associated with poorer disease-free survival ($p < 0.0001$), but not disease-specific survival ($p = 0.164$) (Fig. 1B and C). The rates of disease-free survival at 10 years were 86.4% and 57.6% in the patients with H19 $\leqslant 0.78$ and > 0.78 ($P = 0.003$). The disease-specific survival rates at 10 years were 91.8% in H19 $\leqslant 0.78$ groups, and 99.2% in > 0.78 groups ($P = 0.096$).

We evaluated the prognostic significance of clinicopathological factors for disease-free survival in the 391 patients without distant metastases at the time of surgery. Age $\geqslant 60$ years, tumor > 4 cm, significant extrathyroid extension (Ex2), clinical lateral node metastasis (cN1b), pN status, aggressive histological type and H19 < 0.78 were all significantly associated with disease free survival (Table 2) . Multivariate Cox proportional hazards model indicated that age $\geqslant 60$ years, extrathyroid extension and H19 < 0.78 was an independent prognostic factors for disease-free survival (Table 2) .

4. Discussion

Prognostic assessment is very important in the management of cancer patients. The age of the patient at the diagnosis, gender, tumor size, extrathyroid expansion, lymph node and distant metastasis, and pathological differentiation of cancer are identified well prognostic factors in thyroid cancer. The most commonly used system may be the TNM classification, which uses age, tumor size, extrathyroid expansion, lymph node and distant metastasis for risk assessment. TNM staging can be assessed preoperatively to help plan patient care. Pathological findings provide additional information for assessing pathological TNM staging. Tumor growth activity is also a very important biological factor. However, none of the above systems used a factor directly related to tumor growth activity for risk assessment.

H19 has been identified as one cancer-related lncRNAs. The high expression of H19 was considered to be correlated with diverse human disorders and cancers. Growing evidences showed that this H19 gene-encoded 2.3 kb lncRNA functioned in tumorigenes and cancer progression. The development of cancer may be promoted by the up-regulated expression of H19 [12]. The high expression of H19 may be a biomarker for cancer prognosis. Numerous studies have shown that expression of H19 in tumor tissues was significant higher than that of in healthy or para-carcinoma tissues [13,14]. And the increased expression of H19 in cancer tissues was frequently positively correlated with advanced clinical stage and inversely correlated with patient's prognosis [13,14].

However, in the present study, we found that expression of H19 was significant lower in PTC tissues

than that of in thyroid benign node tissues. Low expression of H19 was associated with age > 60, tumor size > 4 cm, aggressive type, extrathyroid extension and pathological lateral node metastasis, suggesting that H19 may serve as a potential biomarker for PTC prognosis monitor.

In the present multivariate logistic regression analysis, the patient's age at surgery was the indicator of disease-free survival. The age $\geqslant 60$ years was significant factors with hazard ratios of 3.84. In addition, the extrathyroid extension and H19 < 0.78 was also an independent prognostic factors for disease-free survival with hazard ratios of 2.36 and 1.92, respectively. These date indicated that expression of H19 may be as a marker of poor prognosis and, thus, a potential therapeutic target for treating PTC patients.

Our finding concluded that H19 could serve as a feasible prognostic biomarker of PTC. The ROC curve analysis also confirmed that expression of H19 could help differentiate patients with PTC from benign nodes with high sensitivity and specificity. Therefore, H19 could be a diagnostic biomarker of PTC. However the specificity of the PTC to differentiate patients with PTC from benigns is still relatively low. If H19 combines clinical markers commonly used in PTC, such as BRAF, TSH, Tg, etc., it may improve the diagnosis rate of PTC, which need further investigation.

In summary, the present study suggests that H19 was a strong prognostic indicator in patients with PTC. It could screen PTC from thyroid benign nodes. This work represents an advance in biomedical science because it shows that H19 could discriminate PTC patients from thyroid benign nodes, so may represent a novel prognostic and diagnostic markers for PTC.

References

[1] S.A. Hundahl, I.D. Fleming, A.M. Fremgen and H.R. Menck, A national cancer data base report on 53,856 cases of thyroid carcinoma treated in the U.S., 1985–1995, *Cancer* **83**(12) (1998), 2638–2648.

[2] J.C. Lee, J.S. Gundara, A. Glover, J. Serpell and S.B. Sidhu, *Oncologist* **19**(11) (2014), 1141–1147.

[3] R.L. Brown, J.A. de Souza and E.E. Cohen, Thyroid cancer: Burden of illness and management of disease, *J Cancer* **2**(3) (2011), 193–199.

[4] E.L. Mazzaferri and S.M. Jhiang, Long-term impact of initial surgical and medical therapy on papillary and follicular thyroid cancer, *Am J Med* **97**(6) (1994), 418–428.

[5] R.M. Tuttle, D.W. Ball and D. Byrd, Thyroid carcinoma, *J Natl Compr Canc Netw* **8**(4) (2010), 1228–1274.

[6] M. Xing, B.R. Haugen and M. Schlumberger, Progress in molecular-based management of differentiated thyroid cancer, *Lancet* **381**(7) (2013), 1058–1069.

[7] I.J. Matouk, N. Degroot and S. Mezan, The H19 non-coding RNA is essential for human tumor growth, *Plos One* **2**(9) (2017), e845.

[8] I.J. Matouk, D. Halle, M. Gilon and A. Hochberg, The non-coding RNAs of the H19-IGF2 imprinted loci: A focus on biological roles and therapeutic potential in lung cancer, *J Transl Med* **13** (2015), 113.

[9] E. Raveh, I.J. Matouk, M. Gilon and A. Hochberg, The H19 long noncoding RNA in cancer initiation, progression and metastasis-a proposed unifying theory, *Mol Cancer* **14**(6) (2015), 184.

[10] X. Lan, W. Sun, W. Dong, Z. Wang, T. Zhang, L. He and H. Zhang, Downregulation of long noncoding RNA H19 contributes to the proliferation and migration of papillary thyroid carcinoma, *Gene* **646** (2018), 98–105.

[11] P. Wang, G. Liu, W. Xu, H. Liu, Q. Bu and D. Sun, Long noncoding RNA H19 inhibits cell viability, migration, and invasion via downregulation of IRS-1 in thyroid cancer cells, *Technol Cancer Res Treat* **16**(6) (2017), 1102–1112.

[12] E. Raveh, I.J. Matouk, M. Gilon and A. Hochberg, The H19 Long non-coding RNA in cancer initiation, progression and metastasis-a proposed unifying theory, *Mol Cancer* **14** (2015), 184.

[13] M.L. Yang, Z. Huang, Q. Wang, H.H. Chen, S.N. Ma, R. Wu and W.S. Cai, The association of polymorphisms in lncRNA-*H19* with hepatocellular cancer risk and prognosis, *Biosci Rep* **38**(5) (2018), 38.

[14] J.S. Chen, Y.F. Wang, X.Q. Zhang, J.M. Lv, Y. Li, X.X. Liu and T.P. Xu, H19 serves as a diagnostic biomarker and up-regulation of H19 expression contributes to poor prognosis in patients with gastric cancer, *Neoplasm* **63**(2) (2016), 223–230.
